# Sexually transmitted infections: old foes on the rise

**DOI:** 10.15698/mic2016.09.522

**Published:** 2016-09-05

**Authors:** Didac Carmona-Gutierrez, Katharina Kainz, Frank Madeo

**Affiliations:** 1 Institute of Molecular Biosciences, NAWI Graz, University of Graz, Humboldtstr. 50, 8010 Graz, Austria.; 2 BioTechMed-Graz, Humboldtstr. 50, 8010 Graz, Austria.

**Keywords:** STI, syphilis, gonorrhoea, chlamydia, trichomoniasis, hepatitis B, herpes simplex virus, human immunodeficiency virus, human papillomavirus, HPV, HBV, HIV

## Abstract

Sexually transmitted infections (STIs) are commonly spread via sexual contact. It is estimated that one million STIs are acquired every day worldwide. Besides their impact on sexual, reproductive and neonatal health, they can cause disastrous and life-threatening complications if left untreated. In addition to this personal burden, STIs also represent a socioeconomic problem, deriving in treatment costs of tremendous proportions. Despite a substantial progress in diagnosis, treatment and prevention, the incidence of many common STIs is increasing, and STIs continue to represent a global public health problem and a major cause for morbidity and mortality. With this Special Issue, *Microbial Cell* provides an in-depth overview of the eight major STIs, covering all relevant features of each infection.

Sexually transmitted infections (STIs) are old acquaintances. For instance, references to the symptoms of gonorrhea, incidentally a term implemented by the Greek physician Galen in the second century 1, can be found in the Bible 1,2. In medieval times, for example, syphilis was one of the most ubiquitous STIs in Europe. Interestingly, at that time it was a rather novel infection for this continent: according to one hypothesis crew members of the first Columbian expeditions to the Americas might have been responsible for importing this STI and originating the first recorded epidemic of syphilis in Europe in 1495 3,4. Indeed, given the fact that STIs are transmitted via the universal and unavoidable act that is sexual practice, their infectious potential is considerable and has remained a threat until the present day. While young adults (age 15-24) are at higher risk for acquiring an STI, the incidence in the older population (age >50) is growing due to limited sexual health services, increased longevity and the availability of sexual performance enhancement drugs 5,6.

Despite public awareness, prevention strategies and medical advances, STIs continue to persist as a major public health problem. Although it seems that they have lost a lot of their terrifying character, STIs do continue to be an important cause of morbidity and mortality worldwide. For instance, the danger of a possible STI transmission makes unprotected sexual intercourse the second most important risk factor for disability and death in low-income nations and the ninth most important in more economically developed countries 7. STIs have a major impact on sexual, reproductive and neonatal health and - if left untreated - may cause disastrous and even life-threatening complications: pelvic inflammatory disease, infertility, cervical cancer, ectopic pregnancy, miscarriage, fetal death, or congenital infections are only some of the common side effects associated with diverse STIs 8,9. Notably, there might exist infection synergies individuals who are positive for one between different STIs 8,9. This has been most extensively studied for the human immunodeficiency virus (HIV) with a number of biological mechanisms having been suggested to account for such synergies beyond high-risk sexual behavior 10,11. Importantly, patients frequently have to cope not only with their physical afflictions but also with the social and psychological consequences of having an STI. Even in the 21st century, STIs are usually viewed as shameful diseases, and affected individuals are still stigmatized and often enough socially condemned. Hence, suffering from an STI can impact people in nearly all parts of their daily life.

STIs are a problem that impacts society beyond the individual realm, representing a huge economic burden for healthcare systems. For instance, the Centers for Disease Control and Prevention (CDC) estimate the number of new STI cases at about 20 million per year in the US and calculate that the costs for the US healthcare systems to treat the eight most common STIs amount to a total of approximately $16 billion US dollars each year 12. It should be noted that this computation also includes lifelong treatment in terms of incurable STIs (e.g. HIV) and therapy costs for STI-related disorders (e.g. HPV-related cancer). Still, the treatment expenses of curable STIs add up to about $740 million per year 12. Taking all aspects together, prevention is indeed better than cure. Precluding transmission is thus the key approach to counteract these infections, as outlined by the WHO in its global health strategy on STIs 8.

Despite the crucial progress that has been made in terms of diagnosis, treatment and prevention (e.g. vaccination), STIs remain the most widespread and hazardous infectious diseases 9. The WHO estimates that worldwide, about one million STIs are acquired every day 13. Although a transient reduction in infection numbers was recorded in the late 1980s - probably caused by changes in sexual behavior obeying to the nascent HIV pandemic - the incidence of STIs is back on the rise since the mid-1990s 14. In Europe, for instance, the overall infection rates for gonorrhea have more than doubled between 2008 and 2014 15 and in 2014, the recorded number of new HIV cases was the highest since reporting began in the 1980s 16. There are many possible reasons for this increase in the occurrence of STIs. Although this might be partly due to the improved diagnostic capability following technical developments, recent changes in the demographic, social and economic status are thought to be determinant for this distressing trend. Among them, a lower rate of marriages concomitant with increasing numbers of lifetime sex partners, delayed childbirth and enhanced population movement (increased travel rates, international migration) represent only some factors that might influence transmission rates 5,14. In addition, at least for bacterial and protozoan STIs, antibiotic resistance is a growing phenomenon that undermines the available drug arsenal currently used to counteract the associated conditions 17. Of note, the reported disease figures probably underrate the actual burden of infection, because of underreporting and the fact that a large number of STIs remain asymptomatic although they are infectious 5,18. The resulting (unintentional) lack of awareness regarding the danger to transfer the pathogen and/or the smugness about the individual risk to acquire an STI also contributes to increased transmission 19.

So far, pathogen transmission via sexual contact is known for more than 30 different bacteria, viruses and parasites 13. Thereof, three bacterial (syphilis, gonorrhea, chlamydia), a protozoan (trichomoniasis) and four viral (human papilloma virus (HPV), herpes simplex virus (HSV), hepatitis B virus (HBV), and HIV) STIs account for the majority of infection cases 8,13. In this Special Issue on STIs, *Microbial Cell* has compiled a series of articles that focus on these eight major STIs, covering all relevant features of each infection: (i) etiology, transmission and protection; (ii) pathology/symptomatology; (iii) epidemiology, incidence and prevalence; (iv) treatment and curability; and (v) molecular mechanisms of infection. Therefore, this Special Issue attempts to provide a comprehensive overview for the specialized and general public, not least shedding light on STIs as the epidemic of colossal magnitude that it is.
